# A large population sample of African HIV genomes from the 1980s reveals a reduction in subtype D over time associated with propensity for CXCR4 tropism

**DOI:** 10.1186/s12977-022-00612-5

**Published:** 2022-12-13

**Authors:** Heather E. Grant, Sunando Roy, Rachel Williams, Helena Tutill, Bridget Ferns, Patricia A. Cane, J. Wilson Carswell, Deogratius Ssemwanga, Pontiano Kaleebu, Judith Breuer, Andrew J. Leigh Brown

**Affiliations:** 1 Institute of Ecology and Evolution, University of Edinburgh, Edinburgh, UK; 2 Division of Infection and Immunity, University College London, London, UK; 3 UCL Great Ormond Street Institute of Child Health, London, UK; 4 Department of Virology, University College London Hospitals NHS Foundation Trust, London, UK; 5 Medical Research Council (MRC)/Uganda Virus Research Institute (UVRI) and London School of Hygiene and Tropical Medicine (LSHTM) Uganda Research Unit, Entebbe, Uganda; 6 Salisbury, UK; 7 London, UK

**Keywords:** HIV, Subtype D, East Africa, Co-receptor, Target-capture sequencing, Historic samples

## Abstract

We present 109 near full-length HIV genomes amplified from blood serum samples obtained during early 1986 from across Uganda, which to our knowledge is the earliest and largest population sample from the initial phase of the HIV epidemic in Africa. Consensus sequences were made from paired-end Illumina reads with a target-capture approach to amplify HIV material following poor success with standard approaches. In comparisons with a smaller ‘intermediate’ genome dataset from 1998 to 1999 and a ‘modern’ genome dataset from 2007 to 2016, the proportion of subtype D was significantly higher initially, dropping from 67% (73/109), to 57% (26/46) to 17% (82/465) respectively (p < 0.0001). Subtype D has previously been shown to have a faster rate of disease progression than other subtypes in East African population studies, and to have a higher propensity to use the CXCR4 co-receptor (“X4 tropism”); associated with a decrease in time to AIDS. Here we find significant differences in predicted tropism between A1 and D subtypes in all three sample periods considered, which is particularly striking the 1986 sample: 66% (53/80) of subtype D *env* sequences were predicted to be X4 tropic compared with none of the 24 subtype A1. We also analysed the frequency of subtype in the envelope region of inter-subtype recombinants, and found that subtype A1 is over-represented in *env*, suggesting recombination and selection have acted to remove subtype D *env* from circulation. The reduction of subtype D frequency over three decades therefore appears to be a result of selective pressure against X4 tropism and its higher virulence. Lastly, we find a subtype D specific codon deletion at position 24 of the V3 loop, which may explain the higher propensity for subtype D to utilise X4 tropism.

**Supplementary Information:**

The online version contains supplementary material available at 10.1186/s12977-022-00612-5.

## Introduction

The main (M) group of HIV-1 viruses that cause AIDS can be categorised into distinct lineages or “subtypes” [64]. Evidence points to the epicentre of the HIV pandemic being Kinshasa [66] in the early part of the twentieth century [24], and it largely remained within the Democratic Republic of Congo for many decades, undergoing substantial recombination [40, 78]. Strong genetic bottlenecks created geographically [25] and phylogenetically distinct subtypes by the 1960s [80] which subsequently spread throughout Africa and into new susceptible populations across the rest of the world. Today we see this footprint in the global subtype distribution which varies considerably across different countries and risk groups [8].

There has been much speculation and interest in the possibility of phenotypic differences between subtypes (see review by [28] that may have contributed to any one subtype’s relative success over another [23]. Disentangling the relative roles of genetic drift and selective adaptation in HIV lineages amongst hosts is difficult, not least because HIV transmission is heterogeneous, and some lineages may simply be amplified into bottlenecks by chance [62]. Furthermore, subtype comparisons are confounded by differences in mode of transmission (e.g. subtype B in men who have sex with men, [14], and viral characteristics that might be under selection, such as infectivity or virulence, are confounded by a range of factors, including host genetics, (particularly HLA types, [41]). Subtype comparisons within the same country, population, or cohort are therefore strengthened by the reduction of these factors [48]. In Uganda subtypes A1 and D have been co-circulating at high frequencies for many decades [82], in both general population cohorts [70] and high risk communities [6, 68], providing a rare opportunity to compare their impact directly.

Co-receptor tropism (the secondary receptor used alongside CD4) can be distinguished in cell-culture where “fast replicating” syncytium inducing (SI) viruses use CXCR4 (X4 tropic), and “slow” non syncytium inducing (NSI) viruses use CCR5 (R5 tropic) [16]. Fast replicating X4 viruses have long been associated with faster CD4 decline [45], and the risk of AIDS progression could be as much as 3.8 × higher [18], which in real terms translates to multiple years of additional lost life. Comparisons of R5 and X4 viruses at the V3 loop where tropism is largely determined (but not exclusively e.g. [73], indicate that positive amino acid charges at positions 11 and 25 are strongly predictive of X4 tropism (the ‘11/25 rule’; [79]). Currently, more sophisticated machine learning models are used to predict co-receptor tropism based on V3 amino acid training data (e.g., *geno2pheno*, [67]).

Subtype D has been shown consistently to progress to AIDS faster compared with other subtypes [10, 21, 37, 38, 42, 43, 69, 76]. It has also been reported that subtype D viruses are more likely to use CXCR4 co-receptors [36, 39, 74, 77], and that individuals infected with subtype D reach higher viral loads more rapidly [2].

HIV sequencing is important for use in detecting drug resistant mutations, but can also provide insights about epidemic size and diversity e.g. [70] or movement between key populations by phylogenetic analysis e.g. [6, 44]. Sequencing in East Africa up until 2013 had been limited mostly to consensus Sanger sequences of partial gene sequences of p24 or gp41 [49], with very little genome sequence data from the twentieth century, although partial *pol* sequencing has recently become more common since the roll out of antiretroviral therapy. The PANGEA project [59] aimed to rectify this for the twenty-first century and has obtained large datasets of near full-length sequences from Africa to provide more detailed phylogenetic information [82].

Samples from serological surveys conducted in early 1986 from hospitals and antenatal clinics in Uganda were re-discovered in storage in 2013 during the relocation of what were then the Public Health England laboratories at Porton Down. Standard clinical *pol* sequencing [11] was attempted with some limited success [82], and amplification and sequencing success with the PANGEA protocol was also limited (unpublished) due to the age of the samples. To overcome barriers in the face of considerable RNA degradation, we used target-capture techniques with baits designed to capture a wide variety of HIV-1M to recover 109 new near full-length and 37 partial genomes. This is a unique population dataset from the early African epidemic, shortly after AIDS was discovered from a decade where few HIV genomes are available, particularly from Africa.

## Methods

### Sample preparation

Serum samples were collected from across Uganda between January and May 1986, including as part of a serological survey of HIV prevalence in different populations [13]. Samples were sent to Porton Down in the UK for antibody testing in 1986 and were subsequently stored there at − 80 °C. After their rediscovery they were passed to the PANGEA project in 2013.

In the current work, 168 HIV positive samples which had been identified by ELISA were RNA extracted with the QIAamp viral RNA mini kit (Qiagen). A target-capture approach [20] developed for samples with low concentrations or degraded RNA virus genomes was adopted. Thus 120 base pair capture baits were designed with an in-house pipeline to target the whole HIV genome, using 2635 reference genomes covering global subtype and CRF diversity (baits licensed to Agilent no. 5191-6709, SureSelectXT CD Pan HIV1). cDNA libraries were constructed with SuperScript IV Reverse Transcriptase (Invitrogen) followed by NEB Second Strand cDNA Synthesis before using the SureSelectXT Target Enrichment System for Illumina Paired-End Multiplexed Sequencing Library. This included a pre-capture PCR step during library preparation; followed by bait hybridization and a capture step with streptavidin beads to enrich for HIV fragments; and a post-capture indexing PCR. Paired end sequencing was carried out with the Illumina MiSeq v2 500 cycle kit.

### Sequence assembly

Trimming, adapter removal, and quality checking of reads was performed with TrimGalore, cutadapt and FastQC [3, 47, 53], using a minimum Phred score of 30. Mapping to reference genomes was done with the Burrow-Wheeler Aligner MEM algorithm [52] and the samtools and bcftools libraries [19], firstly to 170 reference genomes (encompassing a wide range of subtype and CRF diversity) to identify the best genotype, and then to the best reference for a single reference assembly. A visual assessment in Geneious Prime 2022.0.1 (www.geneious.com) was carried out to check for good coverage across the genome, or any dips that might indicate an inter-subtype recombinant sequence. If this was the case, the multi-reference BAM files were examined, or an alternative de-novo assembly with HAPHPIPE and SPAdes was attempted [5, 29]. Either the single reference assembly (or de-novo assembly if improvement could be found) was then fed into the HAPHPIPE framework for fine tuning with three rounds of iterative improvement. Coverage statistics and vcf files were produced for each and finally a consensus sequence with a minimum of 10 × coverage at every base pair position was generated using GATK [54] within HAPHPIPE.

### ‘Intermediate’ and ‘modern’ datasets

In addition to the newly generated ‘historical’ dataset, a collection (n = 46) of genomes from the Rakai district (Uganda) in 1998 and 1999 provided an ‘intermediate set’ [34], whilst a ‘contemporary set’ was taken from the MRC/UVRI PANGEA genome collection (n = 465) sampled in Central Uganda between 2007 and 2016 (described fully in [30].

### Subtyping and co-receptor prediction

All genomes were subtyped with the full genome version of SCUEAL [46]. All sequences were subjected to co-receptor prediction using the *geno2pheno* co-receptor tool [67] first by aligning the V3 loop by eye and extracting the amino acid sequence in Geneious Prime 2022.0.1 (www.geneious.com). The inter-subtype recombinant genomes (unique recombinant forms; URFs from all three datasets with a clear A1 or subtype D majority (over 70% the length of *env* as determined by SCUEAL breakpoints and clearly covering the V3 loop) were included. Subtype level consensus amino acid sequences were found and Shannon’s entropy of the two were calculated then compared with the Entropy-Two tool from the Los Alamos Database (https://www.hiv.lanl.gov/content/sequence/ENTROPY/entropy.html).

To investigate the origin of a deletion at position 24 in the V3 loop, additional data from the oldest subtype B and D envelope sequences were obtained from the Los Alamos National Laboratory (www.hiv.lanl.gov) for comparison. A BEAST [72] phylogeny was constructed (see [31], and an ancestral state reconstruction for presence or absence of the position 24 deletion by parsimony was then carried out with the R package ‘castor’ [71] and plotted in ggtree [83].

## Results

### Historical sequences

HIV specific baits were used in a target-capture step to enrich HIV genetic material before Illumina MiSeq sequencing to generate a paired-end read dataset of 109 near full-length consensus sequences with a minimum of 10 × coverage at every position. In addition to these near full-length consensus genomes (> 8000 bp from *gag* to *nef*), 37 partial sequences (> 1000 bp) were generated (a 65% genome recovery success from 168 samples, or 87% partial sequence recovery, Additional file 4: Table S1).

**Fig. 1:**
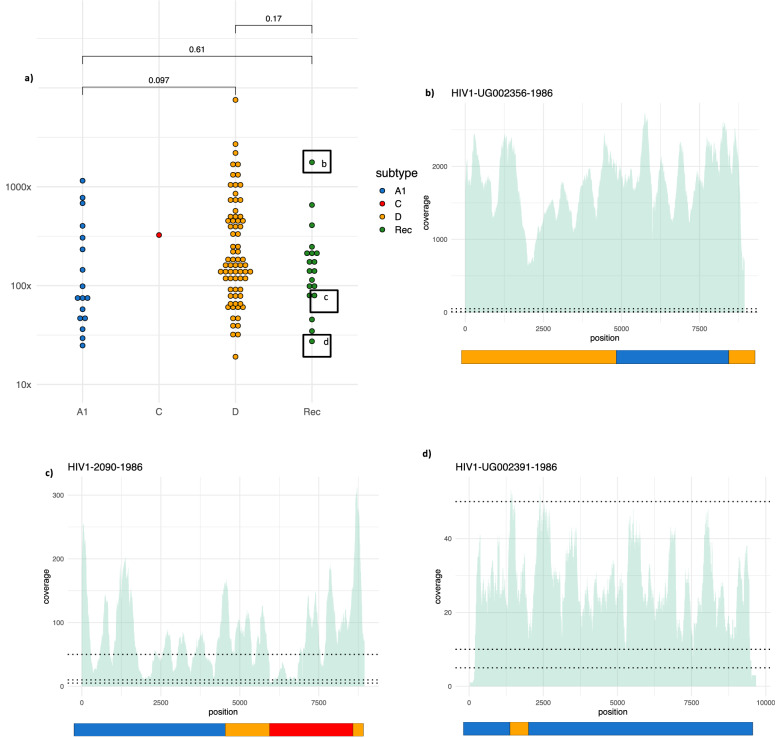
Average read coverage across the genome for each genome assembly, and three example coverage plots of inter-subtype recombinant genomes from historical samples. Panel **a** gives the average coverage per genome assembly by subtype (with base 10 scale), which shows no significant difference between subtypes A1 and D, or between either subtype and the inter-subtype recombinants. Panel **b** shows the coverage plot of the inter-subtype recombinant with the highest average coverage (× 1769), panel **c** shows the only A1,C,D inter-subtype recombinant genome (average × 80), and **d** the lowest coverage inter-subtype recombinant (average × 27). The dotted horizontal lines show 5 × , 10 × and 50 × and the SCUEAL designated subtype and breakpoints are shown as schematic bars underneath each plot (subtype A1 in blue, D in orange, and C in red)

Average coverage spanned from × 27 to × 1769, with no significant difference found between subtypes or between subtypes and inter-subtype recombinants (Fig. 1). This method is considerably more sensitive than without the target-capture step; in 2014 some of these samples were subjected to the PANGEA protocol [27] with modest success, generating 5 near full-length genomes and 17 partial genomes, (a success rate of 5% and 22% respectively from a 96-sample plate; data not shown).

Of the 109 consensus genomes, 90 had some basic location information. The majority are from the “Central” region (n = 55) which includes Kampala and hospitals within Kampala, Rubaga (n = 12), Mulago (n = 8) and Nsambya (n = 7), and unidentified antenatal clinics (n = 2). A further 31 genomes were recovered from Kitovu Hospital (Masaka District), 4 from Lacor hospital in Gulu in northern Uganda, one from a hospital in Jinja (80 km East of Kampala) (see Additional file 5: Table S2 and map of Uganda in Additional file 1: Fig. S1).

### Change in subtype frequency

**Fig. 2:**
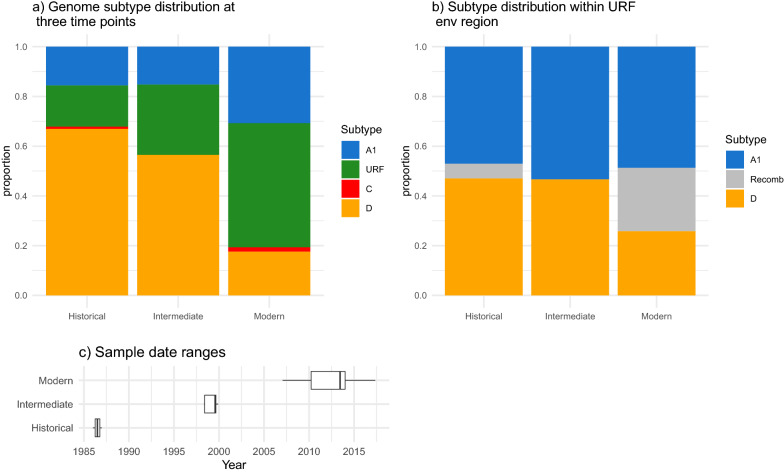
**a** Ugandan subtype distribution from full-genome sequences at three time periods. Bars show subtype proportions in the 1986 ‘historical’ genome set described here n = 109, the ‘intermediate set’ from Harris et al*.* (n = 46), and the ‘modern’ dataset from PANGEA (n = 465), **b** the subtype within URF envelope region over the same three time periods, and **c** the sampling date range of each period as box plots

The overall subtype distribution of the 109 historical 1986 genome set was as follows: 73 subtype D (67.0%); 17 subtype A1 (15.6%); 1 subtype C (0.9%) and 17 inter-subtype recombinants composed of A1, D (15.6%) and 1 composed of A1, C and D (0.9%). All inter-subtype recombinants had a unique recombination pattern (Additional file 2: Fig. S2). Subtype D was the most prevalent in all sampling locations, but particularly prevalent in Kitovu Hospital in Masaka, 130 km to the southwest of Kampala where 27/31 (87%) of genomes and 3/5 (60%) partial genomes were subtype D. The SCUEAL designated subtype distribution for the ‘intermediate’ genome dataset was 26 D (56.5%); 7 A1 (15.2%); and 13 inter-subtype recombinants containing A1, D, and C (28.2%). The ‘modern set’ had the distribution: 82 D (17.6%); 3 C (0.6%); 143 A1 (30.8%) and 232 inter-subtype recombinants (49.9%). The proportional change of genome level subtype over the three periods is illustrated in Fig. 2a. Combining other subtypes with recombinants, the relative frequencies of A1 and D, are significantly different in these three time periods (χ^2^ = 122.68, df = 4, p < 0.0001), and show a significant linear trend for reduction in subtype D genome frequency over time (Cochran Armitage, Z = − 10.861, p < 0.0001).

Furthermore, the frequencies of subtypes A1 and D within the URF envelopes were also assessed (Fig. 2b). The majority subtype within the envelope region of all URFs was determined based on SCUEAL-estimated breakpoints. A threshold of 70% over the length of the *env* gene, including the V3 loop, was used to classify *env* as D, A1, or a recombinant. The relative frequency of subtype D also falls in the URF envelope region over time (Cochran Armitage, Z = − 1.9225, p = 0.027), and considering all time periods together, there were many more URFs with subtype A1 envelopes (n = 131), than URFs with subtype D envelopes (n = 76), significantly different to the expected frequency from the genome level (χ^2^ = 45.973, df = 3, p < 0.0001).

### Co-receptor usage

The machine learning application *geno2pheno* [51] was used to predict virus co-receptor tropism of all V3 sequences in the three datasets (Additional file 6: Table S3). Adopting a 5% false positivity rate threshold, there is a significantly higher proportion of CXCR4 coreceptor usage by subtype D compared with A1 during all three periods (Table 1). Of the historical set, 66% (53/80) of subtype D envelope sequences were predicted to be X4 tropic whilst none (0/24) were predicted to be X4 tropic for the A1 sequences (χ^2^ = 29.8, df = 1, p < 0.0001). Of the intermediate genomes, 33% (11/33) of subtype D were X4 tropic compared with none (0/13) of subtype A1 (χ^2^ = 4.01, df = 1, p = 0.04), and of the modern day 49% (70/143) subtype D were X4 tropic, compared with 5% (13/256) for subtype A1 (χ^2^ = 104.5, df = 1, p < 0.0001).

**Table 1:** Co-receptor tropism predictions for subtypes D and A1 adopting the 5% false discovery rate from *geno2pheno*. Distinction is made between V3 sequences from genomes containing only one subtype and URFs

Subtype	Historic 1986			Intermediate 1998/9			Modern 2007-2016		
	X4	R5	Proportion X4	X4	R5	Proportion X4	X4	R5	Proportion X4
D (genome)	46	26	53/80 (66%)	9	17	11/33 (33%)	44	38	70/143 (49%)
D env (URF)	7	1		2	5		26	35	
A1 (genome)	0	16	0/24 (0%)	0	5	0/13 (0%)	5	136	13/256 (5%)
A1 env (URF)	0	8		0	8		8	107	
Other	0	1	0%	0	0	0%	5	55	8%
Total	53	52	50%	11	35	24%	88	371	19%

### Subtype specific differences in V3 loop at the amino acid level

We used the Los Alamos Entropy-Two tool which uses randomisation with replacement to test for differences in entropy between subtypes. In total, 14 positions were significantly more entropic in Subtype D than A1 (including the crucial positions of 11 and 25), while three sites were more entropic in subtype A1 than D (positions 19, 22, and 24), see Table 2a.

**Table 2:**
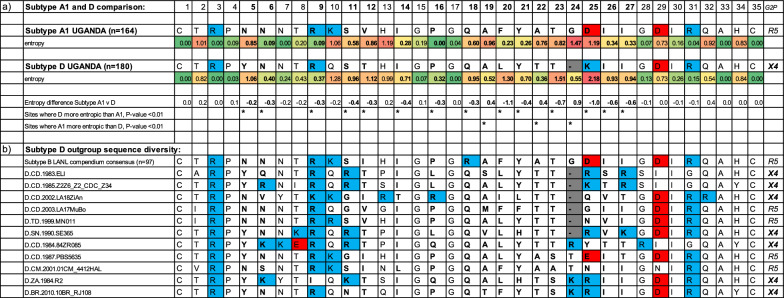
**a** Consensus V3 amino acid sequences of subtypes A1 and D from Uganda with pairwise entropy comparison at each site, and **b** V3 sequences of outgroup sequences to subtype D in Uganda

The Entropy-Two tool from the Los Alamos National Laboratory database was used to compare Shannon’s Entropy at each codon position (indicating variability at each position). Sites with significantly different (p < 0.01) entropy between the subtype A1 consensus and the subtype D consensus are highlighted in bold. Positively charged amino acids (K, Lys) and (R, Arg) are shown in blue, while negatively charged amino acids (D, Asp) and (E, Glu) are shown in red, *geno2pheno* predictions are shown to the right

The consensus length of subtype A1 was 35 codons, whilst that for Subtype D was 34 codons, due to a deletion at position 24 in the majority of both historic (94%; 68/72), and modern-day, (90%; 73/81) subtype D. Whilst the deletion 24 is found in the vast majority of Ugandan subtype D sequences, it is found only in some subtype D outgroup sequences, and not found in the Subtype B consensus (Table 2b). By mapping this deletion onto a subtype B/D phylogeny, we suggest that a deletion arose before the introduction of subtype D in Uganda, but also independently in some other subtype D lineages (see ancestral state reconstruction in Additional file 3: Fig. S3). In the ‘modern’ subtype D dataset, there are a number of additional changes in the V3 loop, including a further codon deletion at position 23 in many sequences, confirming a distinctive difference in the behaviour of this region of *env* in this subtype (see alignment files in Additional files 7 and 8).

## Discussion

Here we describe a population sample of 109 HIV genomes from the early stages of the epidemic in Uganda, and a period where very few HIV genomes are available globally. Most sequences from the early years of the epidemic are now retrospectively obtained by amplification of material from preserved serum or tissue. For example, the oldest sequence fragment to date (ZR59 from the DRC) was obtained from a 1959 plasma sample, but unfortunately, only a few hundred base pairs were sequenced [84] due to its degraded nature, and the limitations of the technology at the time of sequencing. Two well-known isolates (MAL and ELI; [1]) were the first full-length genome sequences generated from African samples obtained contemporaneously, but following passage in cell culture, which would have rapidly accumulated lab-induced changes [58]. We show here that target-capture with next generation sequencing can work well on highly degraded serum samples from over 30 years ago, and without cell passage induced errors. Yamaguchi et al. [81] have also successfully employed similar target-capture methods, obtaining genomes from a wide range of subtypes including from 1987 and the DRC. More recently “jackhammer” techniques recovered a 1966 genome sequence of a subtype C virus, where target-capture methods failed [33]. New sequencing technologies now mean that we are increasingly limited more by the availability of preserved virus material rather than method sensitivity to recover sequences from old samples.

In this historical dataset, most genomes are ‘pure’ subtypes, consistent with the sample being taken during an early point in the Ugandan epidemic when the two subtypes had not co-existed for very long. However, we do find 18 inter-subtype recombinant forms, all of which have a unique pattern, representing at least 18 independent co-infection or super-infection events with different subtypes. Dual infection and recombination between these two subtypes was therefore occurring well before 1986. There is now an extremely high prevalence of unique recombinant forms in Uganda [12, 30, 50], without any evidence of a major circulating recombinant form. This is not unexpected within a generalised epidemic of such large scale and network complexity involving two subtypes at similar prevalence [7, 63], which has not experienced any obvious bottlenecks.

Ugandan cohort studies have been of global interest because unusually, the generalised epidemic provides a natural experiment for directly comparing the phenotypes of two distinct HIV subtypes. These cohort studies have consistently found subtype D to be more virulent than subtype A1, with faster drops in CD4 counts and more rapid progression to AIDS [38, 42, 69]). A faster rate of progression in Subtype D has been confirmed in neighbouring Tanzania [76] and in the UK [21].

There is an extensive literature on the subject of differences in virulence between viral strains, often framed in terms of viral load [9, 26, 35]. Viral load is a well-known predictor of HIV virulence [56]. However, cohort studies often report no significant difference in viral load between subtypes e.g. [10], and it appears that differences in viral load cannot explain differences in mortality risk between subtypes A1 and D [4, 55], suggesting that the “subtype D effect” contributes to virulence even after accounting for differences in viral load [22].

Like viral load, co-receptor usage is also well known to be associated with virulence in HIV [45, 65]. We found a significant co-receptor usage difference between subtypes D and A1, confirming what has been previously reported by studies with smaller sample sizes [36, 39]. Any observation made about co-receptor changes over time at the population level would be confounded by the disease stage of patients, since co-receptor switching is associated with advanced disease stage [16, 45], and many of the 1986 patients would have been experiencing severe AIDS, while many of the modern patients had access to antiretroviral therapies. Taking each of the three time points independently however, we found consistently that subtype D is more likely to be X4 tropic than subtype A1. This difference was particularly stark in the 1986 dataset where 66% (53/80) of subtype D envelopes had X4 tropic viruses compared to 0/24 subtype A1 envelopes. The high proportion of X4 tropism in subtype D may not be surprising given that the majority of the 1986 samples came from late-stage AIDS patients in hospitals, but this was true of *both subtypes*, and none of subtype A1 had an X4 tropism prediction.

Uganda is one of the best sampled countries in East Africa, and these are some of the largest African HIV genome datasets available, but even so, the data here represent only a tiny fraction of the Ugandan epidemic. Additional samples would lend more power to our findings, particularly if they could be stratified into regions and risk groups which are subject to some heterogeneity by subtype (see [6, 68, 70]. Any subtype specific amplification bias can be assumed absent in the historical dataset, since baits were designed with all HIV-1M diversity (2635 reference genomes) and there was no significant difference in read depth between the two subtypes. In the modern dataset, the near full-length genomes and partial genomes had a comparable subtype distribution [30] again suggesting the absence of preferential subtype specific amplification. For the intermediate [34] dataset however, samples underwent cell passage before nested PCR, which may have preferentially amplified X4 viruses and introduced artefacts [57, 75].

Previously, a change in relative proportion of the two subtypes has been shown using sequence fragments of *gag* and gp41 coding regions in a single district (Rakai), between 1994 and 2002 [17] and also between 1993 and 2012 [49]. We support these findings by showing an even more dramatic drop in subtype D, in near full-length genomes, sustained over a longer time period (1986–2016), and over a wider geographical area. All HIV subtype D genes decreased in frequency over time, but this was particularly true in *env*. We looked at URF genomes containing either A1 or D *env* segments and found that subtype D was under-represented compared to subtype A1 at the genome level. This suggests selection has acted with the help of recombination to preferentially include V3 loops with a higher propensity to be R5 tropic (subtype A1) over those with a propensity to be X4 tropic (subtype D) in URFs.

Finally, we show a subtype specific difference at the amino acid level of subtype D, where subtype D has higher entropy at many positions including the key positions 11 and 25, and find a deletion at position 24 which was likely present during the bottleneck of the expansion of subtype D into Uganda from the DRC. This deletion does not always confer X4 tropism, and the R5 phenotype is still predicted by *geno2pheno* for many sequences with the deletion. Instead, we propose that the inherited “sequence space” of subtype D alters the mutational pathways available, pre-disposing subtype D to X4 tropism since there are a multitude of diverse mutational pathways that V3 loops can take to “switch” from R5 to X4 during the course of infection [61]. Interestingly, some authors have reported that the reverse is true for subtype C: which has a lower propensity to utilise CXCR4 [60], because it requires additional mutations to reach X4 tropism [15].

In the 1990s, Uganda mounted a concerted national effort from the highest levels in government down to grass roots which helped to encourage large scale behavioural changes [32]. Once the epidemic was no longer growing, HIV variants would have come under a selective pressure to lengthen the time to AIDS, thereby increasing their effective reproduction and expanding the exposure window [26]. We propose that selection acted against viruses most likely to encode the X4 phenotype, and favored those with the R5 phenotype. Differences in co-receptor switching propensity is therefore a very compelling explanation for the dramatic reduction in subtype D over time, and its association with more rapid disease progression as reported by various Ugandan cohort studies from the twentieth century.

## Supplementary Information

**Additional file 1: Fig. S1.** Map of Uganda showing the largest towns and cities including the sampling locations Kampala, Masaka, Jinja, and Gulu.

**Additional file 2: Fig. S2.** SCUEAL assessment of the 18 inter-subtype recombinants from historical samples. All had a unique recombination pattern. Subtype D fragments shown in orange, A1 in blue, subtype C in red.

**Additional file 3: Fig. S3.** A phylogenetic tree of subtype D (and outgroup subtype B) constructed with *env *and BEAST. Tip labels show presence or absence of a deletion at position 24 in the V3 loop, and an ancestral state reconstruction using parsimony is mapped onto the tree nodes.

**Additional file 4: Table S1.** Information about the 109 genomes and 37 partial sequences including SCUEAL subtype, read depth, location, and Genbank numbers

**Additional file 5: Table S2.** Frequency of HIV genomes by subtype recovered from each sampling location.

**Additional file 6: Table S3.** Amino acid sequences of all V3 loops from all three datasets and *geno2pheno *predictions.

**Additional file 7: Subtype A1 **fasta file alignment of V3 sequences at amino acid level.

**Additional file 8: Subtype D **fasta file alignment of V3 sequences at amino acid level.

## Data Availability

Near full-length genome consensus sequences have been deposited in GenBank (numbers OP039379:OP039487), and partial sequences (OP39488:OP039526), and read data are available on request. SCUEAL (full-length genome version) is available from (https://github.com/veg/hyphy-analyses).
