# Supplementary figures and images for: A large population sample of African HIV genomes from the 1980s reveals a reduction in subtype D over time associated with propensity for CXCR4 tropism
Source: Retrovirology. 2022 Dec 13;19:28. doi: 10.1186/s12977-022-00612-5 (PMC9746199; doi:10.1186/s12977-022-00612-5)

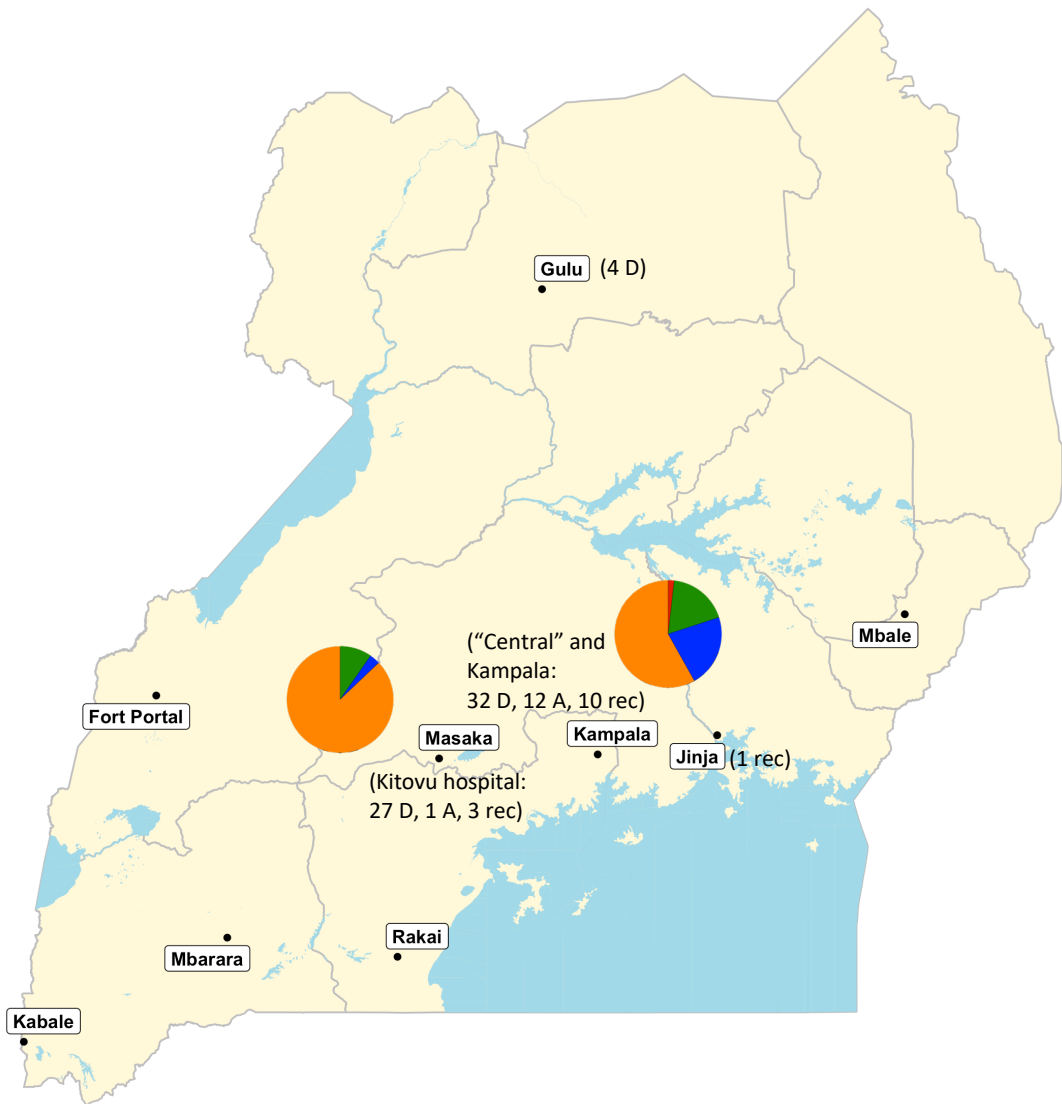

Supplement: Supplementary file 1 — Additional file 1: Fig. S1. Map of Uganda showing the largest towns and cities including the sampling locations Kampala, Masaka, Jinja, and Gulu. [file 12977_2022_612_MOESM1_ESM.pdf]
