# Supplementary material for: A large population sample of African HIV genomes from the 1980s reveals a reduction in subtype D over time associated with propensity for CXCR4 tropism
Source: Retrovirology. 2022 Dec 13;19:28. doi: 10.1186/s12977-022-00612-5 (PMC9746199; doi:10.1186/s12977-022-00612-5)
Supplement: Supplementary file 2 — Additional file 2: Fig. S2. SCUEAL assessment of the 18 inter-subtype recombinants from historical samples. All had a unique recombination pattern. Subtype D fragments shown in orange, A1 in blue, subtype C in red. [file 12977_2022_612_MOESM2_ESM.pdf]

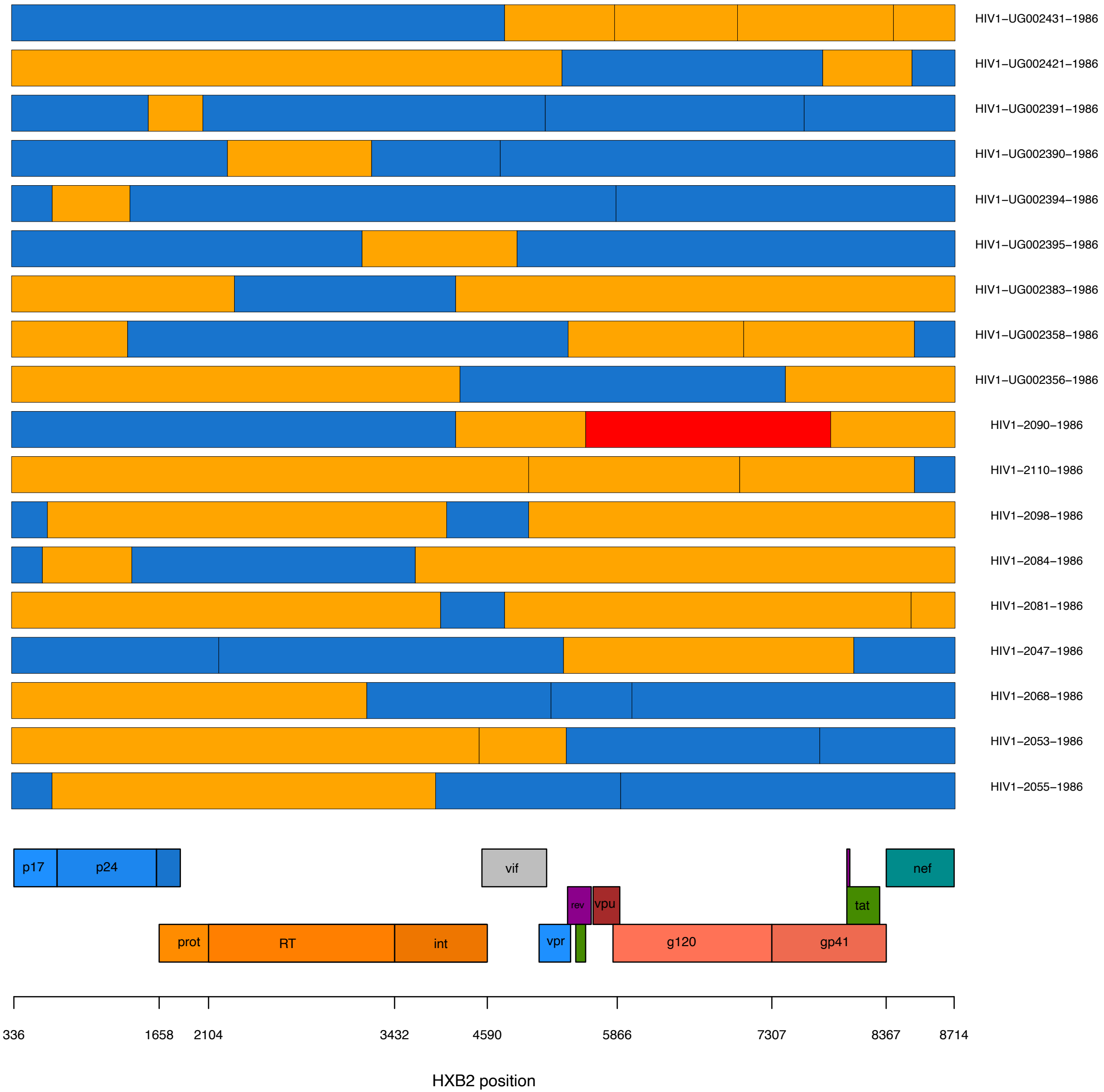
HIV1–UG002431–1986
HIV1–UG002421–1986
HIV1–UG002391–1986
HIV1–UG002390–1986
HIV1–UG002394–1986
HIV1–UG002395–1986
HIV1–UG002383–1986
HIV1–UG002358–1986
HIV1–UG002356–1986
HIV1–2090–1986
HIV1–2110–1986
HIV1–2098–1986
HIV1–2084–1986
HIV1–2081–1986
HIV1–2047–1986
HIV1–2068–1986
HIV1–2053–1986
HIV1–2055–1986
p17
p24
vif
nef
rev
vpu
tat
prot
RT
int
vpr
g120
gp41
336
1658
2104
3432
4590
5866
7307
8367
8714
HXB2 position
