# Supplementary material for: A large population sample of African HIV genomes from the 1980s reveals a reduction in subtype D over time associated with propensity for CXCR4 tropism
Source: Retrovirology. 2022 Dec 13;19:28. doi: 10.1186/s12977-022-00612-5 (PMC9746199; doi:10.1186/s12977-022-00612-5)
Supplement: Supplementary file 3 — Additional file 3: Fig. S3. A phylogenetic tree of subtype D (and outgroup subtype B) constructed with env and BEAST. Tip labels show presence or absence of a deletion at position 24 in the V3 loop, and an ancestral state reconstruction using parsimony is mapped onto the tree nodes. [file 12977_2022_612_MOESM3_ESM.pdf]

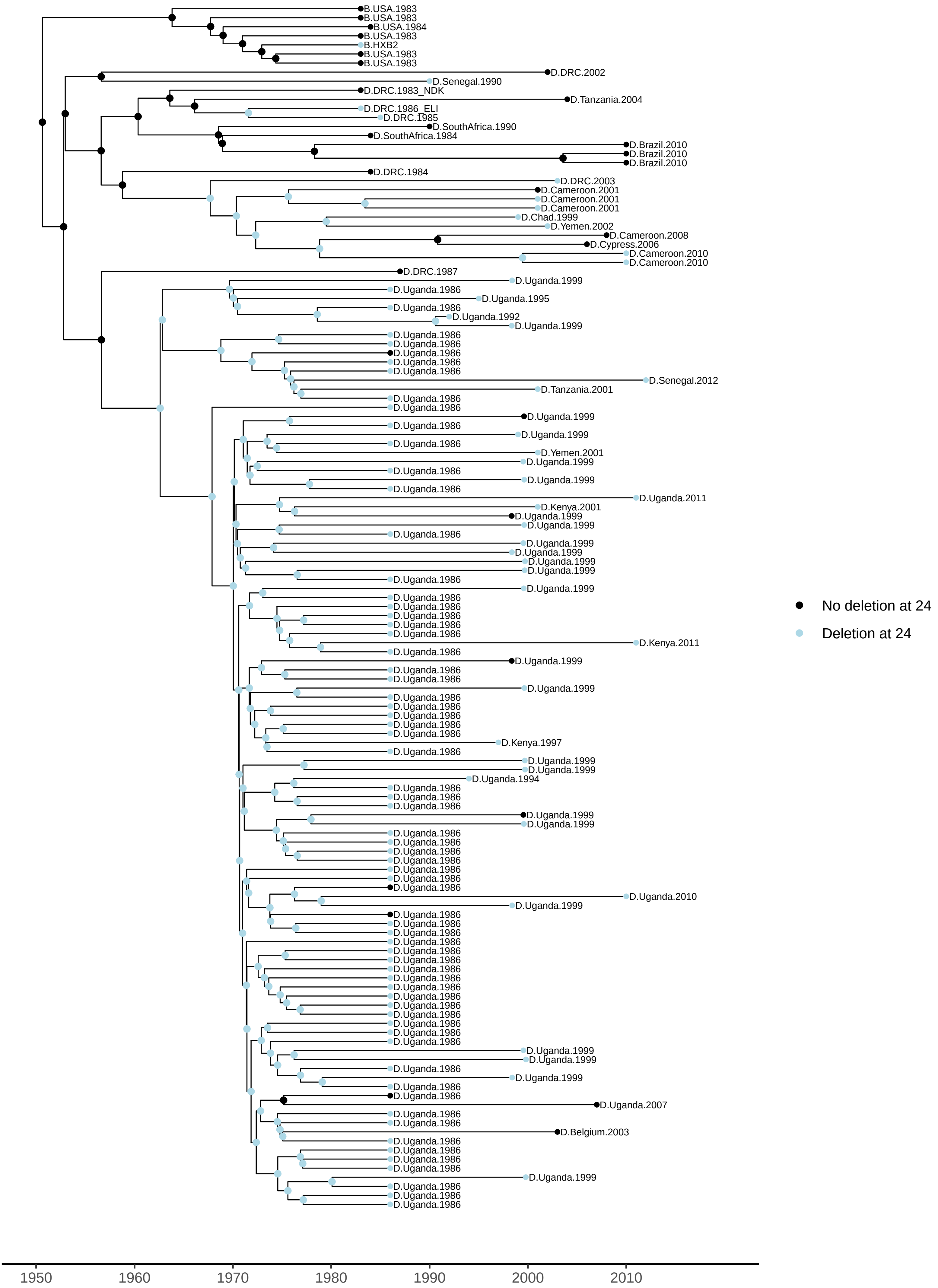
B.USA.1983
B.USA.1983
B.USA.1984
B.USA.1983
B.HXB2
B.USA.1983
B.USA.1983
D.DRC.2002
D.Senegal.1990
D.DRC.1983_NDK
D.Tanzania.2004
D.DRC.1986_ELI
D.DRC.1985
D.SouthAfrica.1990
D.SouthAfrica.1984
D.Brazil.2010
D.Brazil.2010
D.Brazil.2010
D.DRC.1984
D.DRC.2003
D.Cameroon.2001
D.Cameroon.2001
D.Cameroon.2001
D.Chad.1999
D.Yemen.2002
D.Cameroon.2008
D.Cypress.2006
D.Cameroon.2010
D.Cameroon.2010
D.DRC.1987
D.Uganda.1999
D.Uganda.1986
D.Uganda.1995
D.Uganda.1986
D.Uganda.1992
D.Uganda.1999
D.Uganda.1986
D.Uganda.1986
D.Uganda.1986
D.Uganda.1986
D.Uganda.1986
D.Senegal.2012
D.Tanzania.2001
D.Uganda.1986
D.Uganda.1986
D.Uganda.1999
D.Uganda.1986
D.Uganda.1999
D.Uganda.1986
D.Yemen.2001
D.Uganda.1999
D.Uganda.1986
D.Uganda.1999
D.Uganda.1986
D.Uganda.2011
D.Kenya.2001
D.Uganda.1999
D.Uganda.1999
D.Uganda.1986
D.Uganda.1999
D.Uganda.1999
D.Uganda.1999
D.Uganda.1999
D.Uganda.1986
D.Uganda.1999
D.Uganda.1986
D.Uganda.1986
D.Uganda.1986
D.Uganda.1986
D.Uganda.1986
D.Kenya.2011
D.Uganda.1986
D.Uganda.1999
D.Uganda.1986
D.Uganda.1986
D.Uganda.1999
D.Uganda.1986
D.Uganda.1986
D.Uganda.1986
D.Uganda.1986
D.Uganda.1986
D.Kenya.1997
D.Uganda.1986
D.Uganda.1999
D.Uganda.1999
D.Uganda.1994
D.Uganda.1986
D.Uganda.1986
D.Uganda.1986
D.Uganda.1999
D.Uganda.1999
D.Uganda.1986
D.Uganda.1986
D.Uganda.1986
D.Uganda.1986
D.Uganda.1986
D.Uganda.1986
D.Uganda.1986
D.Uganda.2010
D.Uganda.1999
D.Uganda.1986
D.Uganda.1986
D.Uganda.1986
D.Uganda.1986
D.Uganda.1986
D.Uganda.1986
D.Uganda.1986
D.Uganda.1986
D.Uganda.1986
D.Uganda.1986
D.Uganda.1986
D.Uganda.1986
D.Uganda.1986
D.Uganda.1986
D.Uganda.1986
D.Uganda.1999
D.Uganda.1999
D.Uganda.1986
D.Uganda.1999
D.Uganda.1986
D.Uganda.1986
D.Uganda.2007
D.Uganda.1986
D.Uganda.1986
D.Belgium.2003
D.Uganda.1986
D.Uganda.1986
D.Uganda.1986
D.Uganda.1986
D.Uganda.1999
D.Uganda.1986
D.Uganda.1986
D.Uganda.1986
1950
1960
1970
1980
1990
2000
2010
No deletion at 24
Deletion at 24
